# Supplementary material for: Investigating the conception of collaborative learning (CL) and student engagement in the acquisition of practical skills (SEPSA) among prospective physical education and sports students
Source: PLoS One. 2024 Feb 15;19(2):e0288568. doi: 10.1371/journal.pone.0288568 (PMC10868738; doi:10.1371/journal.pone.0288568)
Supplement: S1 File — (PDF) [file pone.0288568.s001.pdf]

Office of the ethical Research Committee

Ref: Ethical: Article9012/2022/EDU/005

Abai Kazakh National Pedagogical University

Dostyk Ave 13, Almaty 050010, Kazakhstan

Phone: +7 7272 21 85 14

June 15, 2022

Subject: Ethical Approval Granted for Research Study

Dear Yerlan Temirkhanov and co-authors,

I am pleased to inform you that your research study titled "**Investigating the conception of collaborative learning (CL) and student engagement in the acquisition of practical skills (SEPSA) among prospective physical education and sports students."** has received ethical approval from Ethics committee of young researchers - Abai Kazakh National Pedagogical University. We have carefully reviewed your research proposal and are satisfied with the adherence to ethical guidelines and principles. We appreciate the detailed attention you have given to ethical considerations, ensuring the protection of participants' rights, privacy, and well-being throughout the study. Your commitment to obtaining informed consent, maintaining confidentiality, promoting voluntary participation, and mitigating potential risks has been duly noted.

We acknowledge the importance of your research objectives and the potential contributions it can make to the physical education and sports field. We believe that your study design and ethical safeguards are in line with the highest standards of research integrity. We kindly remind you to proceed with the study strictly adhering to the approved research protocol and the ethical considerations outlined in your proposal. Any deviations from the approved plan should be promptly communicated to the Ethics Committee for further review and approval. Should any ethical concerns or unforeseen issues arise during the course of your study, we request that you inform the Ethics Committee immediately. Your continued compliance with ethical guidelines will ensure the validity and credibility of your research findings.

On behalf of Ethics committee of young researchers - Abai Kazakh National Pedagogical University, I extend my congratulations on receiving ethical approval for your research study. We look forward to the successful completion of your study and the valuable insights it will contribute to the field. If you have any questions or require further assistance, please do not hesitate to contact us. Once again, we congratulate you on this achievement and wish you every success in your research endeavor.

Sincerely,

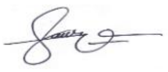

Member Secretary
ETHICS COMMITTEE (EC)

Karim Baigutov

In charge Ethical recommendations and approval committee
